# The Role of Dogs in the Relationship between Telework and Performance via Affect: A Moderated Moderated Mediation Analysis

**DOI:** 10.3390/ani12131727

**Published:** 2022-07-04

**Authors:** Ana Junça-Silva, Margarida Almeida, Catarina Gomes

**Affiliations:** 1 ISCTE–IUL, Business Research Unit, Lisbon University Institute, Avenida das Forças Armadas, 1649-026 Lisboa, Portugal; 2 Instituto Politécnico de Tomar, Escola Superior de Gestão de Tomar, Quinta do Contador, Estrada da Serra, 2300-313 Tomar, Portugal; margarida.almeida@ipt.pt; 3 CIPES, Centro de Investigação em Política, Economia e Sociedade, Universidade Lusófona de Humanidades e Tecnologias, Campo Grande, 376, 1749-024 Lisboa, Portugal; catarinagomes04@gmail.com; 4 Escola de Ciências Económicas e das Organizações, Universidade Lusófona de Humanidades e Tecnologias, 1749-024 Lisboa, Portugal; 5 TRIE: Centro de Investigação Transdisciplinar Para o Empreendedorismo e Inovação Ecossistémica, Universidade Lusófona de Humanidades e Tecnologias, 1749-024 Lisboa, Portugal; 6 CICPSI, Faculdade de Psicologia, Universidade de Lisboa, Alameda da Universidade, 1649-013 Lisboa, Portugal

**Keywords:** pet attachment, telework, positive affect, self-reported job performance, pet closeness, dog ownership

## Abstract

**Simple Summary:**

In this research, we conducted a study with an overall sample of 401 individuals to test a mediating model between telework, positive affect, and self-reported job performance. Additionally, we analyzed whether dogs’ physical closeness and emotional attachment would moderate this mediating path. The results showed that telework was significantly and positively related to positive affect, which in turn, increased self-reported job performance. Moreover, the mediation model was moderated by the dog’s physical closeness while working and emotional attachment to them, in such a way that the relationship between telework on self-reported job performance, via positive affect was strengthened when the owner‘s physical and emotional closeness to their dogs was higher. In sum, telework might be an efficient strategy to improve performance among employees who have dogs at home, because working with them nearby, when emotionally attached to them, are factors that enhance the individual‘s self-perceived performance in telework.

**Abstract:**

Although there is evidence that pets may help individuals facing significant daily stressors, and that they may enhance the well-being of their owners, little is known about the benefits of pets for job performance. Since the COVID-19 pandemic crisis, teleworking was a strategy implemented in many countries to reduce the virus widespread and to assure organizational productivity. Those who work from home and who own pets may work close to them. Based on the conservation of resources theory, this study aimed to analyze whether positive affect mediated the relationship between telecommuting and self-reported job performance and if psychological and physical closeness to the pet would moderate this relationship in such a way that it would be stronger for those who worked closer to their pet, and who were more emotionally attached to them. For this study, we collected data from 81 teleworkers who did not own pets, and from 320 teleworkers who owned pets. Both answered an online questionnaire. Findings: Results from the study showed the existence of significant differences between those who owned and who did not own pets regarding positive affect and performance, in which those who owned pets reported higher levels of positive affect and self-reported performance and perceived telework more positively. Moreover, positive affect mediated the relationship between telework and self-reported job performance. Furthermore, emotional and physical closeness moderated the mediating effect. This study contributes to a better understanding of the human-animal interaction and how pets can be a personal resource able to change their owners‘ affective experiences and job performance while they are working from home. The findings demonstrate that telework may be a suitable organizational strategy for pet-owners.

## 1. Introduction

“Dogs do not need space; they just need to be nearby you!” (Jesse Koz & Shurastey).

The quote above represents that dogs and the bonds they have with their owners are a personal resource for them, inclusive while working [1]. The conservation of resources theory (COR) [2] argues that individuals who possess greater resources are less vulnerable to resource loss and more capable of resource gain [3]. Thus, individuals higher in personal resources (e.g., pet closeness) are in a better position to invest resources that, in turn, may result in positive outcomes such as performance [4].

Telework is a work arrangement that allows employees to work from a remote location [5]. Since the pandemic crisis of COVID-19, organizations were forced to implement it to survive and reduce the chance of spreading the virus. Telecommuting has been found to be positively associated with autonomy and flexibility [6] that in turn appears to enhance job satisfaction [7] and positive affect [8].

Despite the correlational evidence of the benefits of dogs for positive outcomes, such as well-being or stress reduction [9], studies exploring their benefits for workplace outcomes are scarce. As such, this study attempts to determine why attitudes toward telecommuting predict the quality of self-reported job performance and when it will occur. Thus, drawing on the COR theory, we propose that pets, conceptualized as a resource, can influence the effects of attitudes towards telecommuting on affect and self-perceived performance, thus moderating the mediating effect.

## 2. The Relationship between Telework, Positive Affect, and Performance

Telework, originally proposed by Nilles in the 1970s, is a work arrangement also recognized as telecommuting or remote work and was originally defined as working from a remote location away from a standard office or work site [5]. It was defined as “working outside the conventional workplace and communicating by way of telecommunications or computer-based technology” [5] (p. 384). Similarly, Fitzer [10] defined telecommuting as a “work arrangement in which employees perform their regular work at a site other than the ordinary workplace, supported by technological connections” [11] (p. 336).

Telework has progressively spread over the last 40 years and has been strongly encouraged by the measures to limit the COVID-19 pandemic [12]. The Global Workplace Analytics (2019 cited in [13]) predicted that over 70% of the workforce will be working remotely (at least five days per month) in the next five years.

Gajendran and Harrison [14] evidenced that “telecommuting is mainly a good thing” (p. 1535) and showed that it was associated with increased perceptions of autonomy at work. Telecommuting also provides freedom and flexibility and offers many benefits such as positive affective experiences [6,7,15].

Positive affect includes brief and multidimensional affective responses to events or changes in the environment and is based on the individual‘s interpretation of these events or contexts [16,17]. Accordingly, positive affect leads individuals to engage in novel and larger behavioral repertoires; and is related to positive behaviors that are important for workers’ performance, such as giving more attention to the tasks at hand [18]. In addition, positive affect serves to build personal resources that help workers to deal effectively with their daily life at work. These resources may encompass physical (e.g., energy), intellectual (e.g., knowledge), social (e.g., empathy), and psychological (e.g., engagement) aspects that buffer against the deleterious effects of daily hassles [19]. Past research suggested that resources are fundamental for individuals dealing with work-related hassles [20] and job demands [21], and for energizing performance [22]. In this regard, resources can include positive behaviors, such as perceived task performance.

There are differences between task and contextual performance [23]. While task performance is related to the core tasks of the individual and therefore is related to the organizational goals (e.g., goal attainment, judgment, and decision-making), the contextual performance is referred to all the activities and behaviours that contribute to the work‘s psychological climate and include, for example, helping colleagues engage in learning.

Working from home might lead to different subjective experiences from individual to individual. For instance, it is likely that many workers worked remotely for the first time, and many of them had no choice. Despite this and based on the social exchange theory and on the broad-and-brand theory, we expect that telecommuting, by promoting flexibility, autonomy and freedom will increase positive affect and this, in turn, will enhance job performance. Thus, we defined the following hypothesis:

**H1.**  *The attitude toward telework will be positively related to self-perceived performance.*

**H2.**  *Positive affect will mediate the positive relationship between the attitude toward telework and self-reported job performance.*

## 3. The Moderating Role of Pet Attachment and Physical Closeness

Human-pet interaction and bonding is an interspecies relationship that is historically old [24]. With respect to this Brickel [25], suggested that animals provide “one highly reliable association in a person’s life ... more consistent and reliable than human-human” (p. 310). Moreover, Bradshaw [26], reinforced that: pets hold a “relationship of mutualism” with their owners; that is, pet owners believe they not only give but receive love and affection from their animals. Likewise, Cusak [27], argued that pets are human confidants with no risk of betrayal. Indeed, pets can create connections through their vivacity and ability to interact with humans. Plus, they are sensitive to the feelings of their owners and change their behavior in certain situations [28,29].

The importance of pets for human life has received some attention. Indeed, pets can provide individuals with many benefits, such as stress reduction and increased well-being [30]. However, these findings are controversial because some studies demonstrated significant positive effects whereas others showed non-significant ones [31,32]. While some authors argued that positive effects on health were non-significant, others demonstrated the opposite. For instance, Dotson and Hyatt [24], showed that pet ownership lowers blood pressure, helps to prevent heart disease, to fight depression, and therefore improves one‘s health. Moreover, pets can increase the mental health of their human owners [33]. Perhaps, this may justify the increased importance attributed to pets.

The number of individuals with pets is increasingly higher than some decades ago [31]; moreover, pets are considered, for many owners, as family members, best friends, companions, or “furry babies” [9]. As the number of pets increases, and as pets take a more central role in the lives of individuals, there is an increased need to consider how having a pet at home might affect an individual‘s work-related outcomes, such as job performance [9]. Although often overlooked, pets cross with organizations in relevant ways. More recently, organizations and managers have acknowledged it and some of them are becoming pet-friendly. For instance, there are organizations that let their workers take their pets to work, for instance, as Amazon or Google. By accommodating pets, organizations promote positive effects for workers, since many of them consider their pets as family members [34]. Many studies demonstrated that having a pet around increases positive affect and the number of prosocial behaviors [35]. Similarly, Wagner and Pina e Cunha [1], found that the presence of pets at work reduced stress, improved communication, and enhanced social cohesion. Moreover, Pina e Cunha et al. [36], stated that in companies where employees may bring their pets to work, problems tend to be addressed openly, and employees have more autonomy, with flexibility for breaks and greater tolerance for failure and errors. Hall and Mills [37] reported that workers who often took their pets to work reported higher work engagement, work-based friendship, and fewer turnover intentions, compared to those who never took their pets to work. Hall and Mills [37] also showed that those who frequently took their pet to work evidenced higher work-related quality of life, general wellbeing, home-work interface, job-career satisfaction, more control at work, and better-perceived working conditions compared to those who never took their pet to work.

One reason why people might benefit from working closer to their pets is that they represent an important source of social support. Hence, several studies showed that higher social support improved psychological and physiological health [38,39], greater self-esteem [38], and higher performance rates [40].

Following the recommendations of the World Health Organization, in March 2020, many governments swiftly enacted states of emergency, involving, for example, mandatory telecommuting, stay-at-home orders, physical distancing requirements, and quarantine measures for exposed individuals [41,42]. As COVID-19 pushes employees to work from home, many employees are working alongside their pets for the first time. Also, due to social distancing measures of the COVID-19 crisis, it is likely that pets become more important for individuals, particularly those who live alone. Pets may have the potential to help individuals cope with the loneliness and anxiety that may come from social distance and isolation that comes from telecommuting, as well as the uncertainness and worry that comes with thinking about COVID-19. This presents a unique context to better understand how working closer to pets may affect employee work behavior and attitudes and to better understand the pros and cons of working alongside pets.

The conservation of resources theory [2] is appropriate in further explaining employees’ personal gains from working closer to their pets. Accordingly, the theory states that individuals who possess greater resources are less vulnerable to resource loss and more capable of resource gain [3]. Thus, when individuals are higher in personal resources (e.g., working close to their pets), they become less vulnerable to resource loss and are in a better position to invest resources into the engagement process and that, in turn, may result in positive outcomes, such as job performance [4].

**Figure 1:**
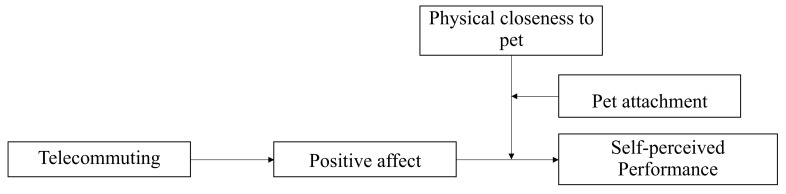
Conceptual model (moderated moderated mediation).

Drawing on COR theory, we propose that pets, conceptualized as a resource, can assist in understanding how people face the effects of working from home, due to the coronavirus, on affect and performance, thus moderating this mediating effect. Specifically, we expect that individuals with higher levels of pet attachment and who work close to their pets will be able to focus on the tasks at hand, improving their self-reported job performance, while working from home (see Figure 1).

**H3.**  *The relationship between the attitude toward telework and self-reported job performance, through positive affect, will be moderated by the physical closeness to pets while working, and by the emotional attachment to them, such that the indirect effect becomes stronger when individuals work closer to their pets (versus not working nearby them) and when they show higher levels of emotional attachment to them (versus lower levels) (moderated moderated mediation).*

## 4. Method

### 4.1. Participants and Procedure

Before conducting the study, this was approved by the ethics committee of the university, thereby we could proceed with the study. We used a non-probabilistic convenience sample as we resorted to participants from our professional networks. We collected data from two groups of teleworkers. One group (*n* = 81) did not own dogs, and the other group owned dogs (*n* = 320). All the participants, from the two groups, were Portuguese individuals transitioning from working at a traditional work location (e.g., an office) to working at home because of the coronavirus and the resultant mandatory confinement.

We collected data through a questionnaire-based survey on the second mandatory confinement due to the COVID-19 pandemic crisis (during February and March of 2021 which was one of the peak periods of COVID-19 in Portugal). Participants from our professional networks were emailed to participate in a study about attitudes at work and toward animals. In order to meet the ethical requirements, we ensured them the anonymity and confidentiality of their responses and we asked them to reply if they agreed to participate. Those who answered the email received another one with the link for the survey. Overall, 450 general questionnaires were distributed among teleworkers, from which 401 agreed to voluntarily participate in the study (response rate: 89.11%).

#### 4.1.1. The Group without Pets

We collected data from 81 teleworkers who did not own pets. The mean age was 32.09 years old (*SD* = 9.48), of which 51% were male and 58% reported being single. The mean organizational tenure was 6.20 years (*SD* = 8.60), and the mean hours worked per week was 40.31 (*SD* = 12.10). On average, the household consisted of 2.5 individuals (*SD* = 1.30) and most of the participants did not have kids at home (73.6%). Participants worked in diverse occupational areas, being the majority in administrative functions (46%), marketing (32%), and teaching (22%).

#### 4.1.2. The Group with Pets

Of the 320 participants with pets, most of them were female (62%), the mean age was 31.87 years old (*SD* = 9.50), and the mean organizational tenure was 5.13 years (*SD* = 7.78). Participants worked in several occupations, being the majority in administrative functions (58%), teaching (32%), and insurance salesman (10%). On average, participants worked about 41 h per week (*SD* = 11.12) and had 1.31 animals (*SD* = 1.31), of which 1.04 (*SD* = 1.22) lived indoor, and 0.30 (*SD* = 0.82) lived outside the house. All participants reported being owners of dogs (100%). On average, the household consisted of 2.82 individuals (*SD* = 1.59) and most of the participants did not have kids at home (68.4%).

### 4.2. Measures

The attitude toward telework was measured with the 17-item E-Work Life Scale [43]. This scale measures four aspects related to the perceived quality of telework experience: effectiveness/productivity (four items; e.g., “When e-working I can concentrate better on my work tasks”), organizational trust (three items; e.g., “My organisation provides training in e-working skills and behaviours.”), the interference between personal and work-life (seven items; e.g., “My social life is not poor when e-working remotely”), and flexibility (three items; e.g., “My work is so flexible I could easily take time off e-working remotely, if and when I want to.”). Participants answered on a 5-point scale (1—totally disagree; 5—totally agree). The Cronbach‘s alpha for this scale was 0.73.

Positive affect was measured with eight items from Multi-Affect Indicator [44] to assess the frequency of daily positive affect experienced at work on that day (e.g., “enthusiastic”). Participants answered on a 5-point scale (1—never; 5—always). The Cronbach‘s alpha was 0.90.

Self-reported job performance was assessed through the 6-item In-Role Performance Scale [45]: “Today, I achieved my job goals”. Items were rated on a 5-point scale ranging from 1 (very little) to 5 (a great deal) (α = 0.84).

The emotional attachment was assessed through the Lexington Attachment to Pets Scale (LAPS) [46]. This scale assesses the perception of individuals towards their pets, and is divided into three dimensions: (1) general attachment (11 items; e.g., “6. I play with my pet quite often.”); (2) people substituting (seven items; e.g., “My pet means more to me than some friends”); (3) animals rights/animal welfare (five items; e.g., “I consider my pet to be part of the family”). Answers were given on a 5-point Likert scale ranging from (1) completely disagree to (5) completely agree. In this study, we only used the first dimension of the scale (α = 0.97).

Physical closeness to pets was measured with four items focused on physical and interaction moments with pets while working. Responses were given on a five-point Likert scale (1—never; 5—always) (e.g., “In telework, I usually take breaks to interact with my pet”; “While you work from home, my pet is close to me when I am working”, “In telework, my pet is not close to me while I work”; “While I am working, I use to interact with my pet”). The Cronbach‘s alpha was 0.87.

Control variables. We used sex and age as control variables. Sex may account for differences in experienced affect [44] and age may account for differences in the perceived experience of telework [43].

Each of the five surveys described above is included in Appendix A.

### 4.3. Data Analysis

Relationships were tested using PROCESS macro 3.1 [47] (in SPSS v. 25), specifically model 4 (mediation) and 18 (moderated moderated mediation). Control variables (age, sex) were entered in Step 1 with telework entered as the independent variable, positive affect as the mediator, and performance as the dependent one (mediating model). Then, physical closeness and emotional attachment to pets were entered as the moderator variables, the products were mean-centered, and bootstrapping (5000 times) was used to provide confidence intervals (moderated moderated mediation).

As both the predictor and the criterion variables were measured at the same time, we took some measures to avoid the issue of common method variance [48]. First, we shuffled the questions of various measures and then used various dummy questions (e.g., I like pets). Second, Harman‘s single factor test was used to assess the common method variance, and it was observed that the single factor accounted for only 22.95% variance, which was much below the standard value of 50% proposed by Podsakoff et al. [49], thus the common method variance issue was not severe for this study.

## 5. Results

### 5.1. Descriptive Statistics and Correlations

Table 1 shows the descriptive statistics and correlations between the variables.

### 5.2. Means Comparison between Groups

Before testing our hypotheses, we analyzed whether there were differences among the variables under study between the two groups of participants (pet owners and non-pet owners). Results showed statistically significant differences for positive perceived effects of telework (*F*_(398)_ = 4.80, *p* < 0.001), positive affect (*F*_(398)_ = 4.27, *p* < 0.01), and performance (*F*_(398)_ = 3.39, *p* < 0.05), suggesting that pet-owners had a more positive perception of telework (*M* = 3.30, *SD* = 0.46 versus *M* = 3.20, *SD* = 0.45) experienced more positive affect (*M* = 3.21, *SD* = 0.54 versus *M* = 3.11, *SD* = 0.73) and showed higher levels of self-reported performance than non-pet owners (*M* = 4.10, *SD* = 0.55; *M* = 3.98, *SD* = 0.45), respectively (see Table 2).

### 5.3. Hypotheses Testing

Hypothesis 1 predicted that the attitude toward telework would be positively related to perceived job performance. The results evidenced that the attitude toward telecommuting was significantly related to perceived job performance (*B* = 0.33, *p* < 0.001). Therefore, the first hypothesis was supported by the data.

Hypothesis 2 expected that the attitude toward telework would positively influence self-reported job performance through positive affect. The results showed a significant indirect effect of positive affect (0.15 with a 95% CI [0.08, 0.22]). Moreover, the relationship between telework and positive affect (*B* = 0.67, *p* < 0.01) and between positive affect and self-reported job performance (*B* = 0.22, *p* < 0.01) were significant. The total effect (*B* *=* 0.33, *p* < 0.01) between the attitude toward telework and self-reported job performance was also significant. After entering positive affect, the effect of the attitude toward telework on self-reported job performance remained significant (*B* = 0.18, *p* < 0.01) suggesting a partial mediating relationship, and thus lending support to hypothesis 2 (see Table 3).

Hypothesis 3 expected that the indirect effect of perceived effects of telework on self-reported performance via positive affect would be moderated by pet physical closeness and emotional attachment, in such a way that the relationship would become stronger for those who were closer (versus distant) and more attached to their pets (versus lower attachment). To test this hypothesis, we followed the recommendations from Hayes [48] to perform the moderated moderated mediation. The results showed that the moderated moderated mediation index was 0.26 (CI 95% [0.02, 0.52]). This means that the indirect effect of telework on self-reported job performance (through positive affect) differs between individuals who work closer to their pets and with different pet attachment levels (see Table 4).

**Figure 2:**
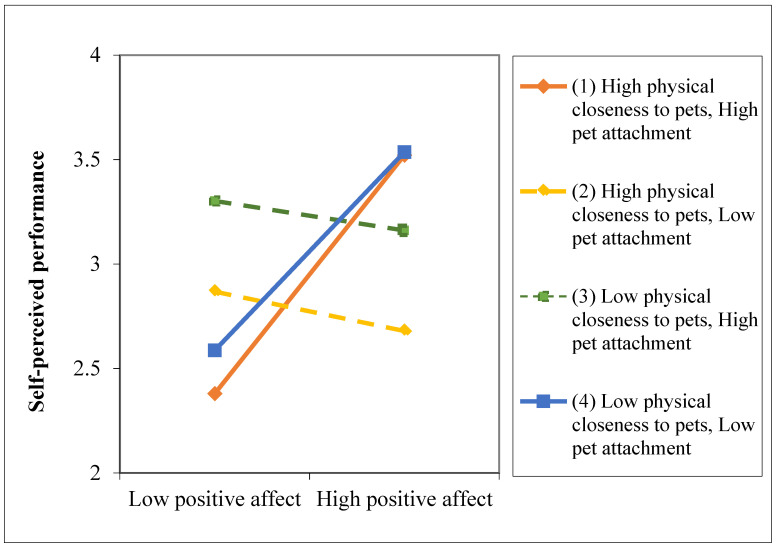
Indirect effect of telecommuting on self-perceived performance through positive affect conditional on pet closeness and attachment.

**Figure 3:**
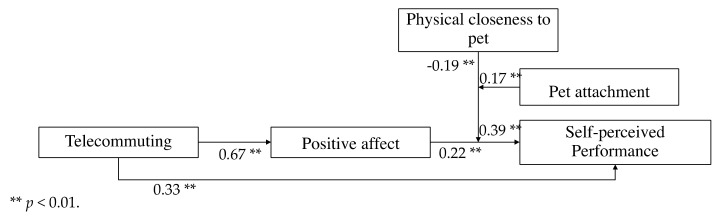
The moderated moderated mediation model with the results.

Then, we followed the suggestion of Hayes [48] to probe the conditional indirect effect. Specifically, we examined the magnitude and significance of the indirect effect of telework on self-reported job performance through positive affect, conditional on physical closeness to pets (at −1SD, mean, and +1SD) for pet attachment levels. The slope analysis showed that the indirect effect was significant for (1) individuals that worked closer to their pets, and had higher attachment levels to them (*B* = 0.20, *p* < 0.01, with CI 95% [0.10, 0.31]), and for (2) individuals who did not work closer to their pets, but whose pet attachment was lower (*B* = 0.07, *p* < 0.01, with CI 95% [0.06, 0.34]) (Figure 2). Thus, the third hypothesis was supported (Figure 3).

## 6. Discussion

The present study examined the role of positive affect on the self-reported job performance of teleworkers highlighting the importance of pet physical and emotional closeness on job performance of them. This study answers the call of studies from [9] to explore the benefits of pets for performance outcomes. Specifically, this study aimed to contribute to understanding the process and the conditions through which telecommuting improves self-reported job performance.

First, the results show that the attitude toward telecommuting is positively associated with self-perceived job performance, that is, while telecommuting workers appear to consider having a positive performance. This result is in line with other studies that have shown a positive effect of working from home on job performance [43].

Second, the results show that the attitude toward telecommuting improves positive affect which in turn enhances self-reported job performance. That is when individuals show higher levels of perceived life quality while teleworking, this tends to positively influence positive affect while working which in turn promotes self-reported job performance. This result is supported from a social exchange perspective. Accordingly, individuals tend to behave by weighing the costs and benefits that they expect to receive (e.g., flexibility) [7]. Thus, individuals who are telecommuters have more flexibility and autonomy at work, raising their feelings of obligation towards their organization, which in turn may enhance their positive affect while working promoting self-reported job performance. Moreover, telework is a model of work characterized by increased levels of flexibility, autonomy, and a sense of control over work [43]. These positive work characteristics trigger more frequently positive affect among workers [8,50], despite the pandemic times being lived. This is evidenced by the job characteristics model [51]. The model has been widely used to determine whether certain core characteristics of jobs (e.g., autonomy) do evoke some affective reactions by workers. Accordingly, autonomy has been consistently related to positive affect and other affective and motivational indicators [52]. Therefore, it is not surprising that telecommuting enhances positive affect at work. Additionally, the broaden-and-build theory suggests that positive affect broadens positive behaviors [15]. Accordingly, positive affect leads individuals to engage in novel and larger behavioral repertoires; and is related to positive behaviors that are important for workers’ performance, such as giving more attention to the tasks at hand [18]. Plus, positive affect builds personal resources that help workers to energize performance [22]. Empirically, there is also evidence of the positive link between telework, positive affect, and self-reported job performance. For instance, Anderson et al. [53], showed that workers showed more positive emotions when they were teleworking when compared to days at the office. Similarly, Abdel Hadi et al. [54], in a diary study developed during the pandemic crisis of COVID-19, showed that individuals in telecommuting experienced fewer negative emotions and better performance rates.

Notwithstanding, this mediating effect seems to be conditional to the physical closeness to pets while working, and to the emotional attachment to them. Specifically, the results show that the mediation is stronger when telecommuters are working closer to their pets, and when they hold an emotional bond with them. That is, when telecommuting, positive affect is more frequent, leading to increases in self-reported job performance, for employees who work closer to their pets, and who demonstrate a high level of attachment to them. The mediation is also significant for individuals who do not work close to their pet, but whose emotional attachment is low. Although it is not a significant decrease, when emotional attachment to pets is high, and work is not being carried out closer to them, self-reported job performance tends to decrease, even after experiencing positive affect. There is evidence that the interaction between humans and their pets arouses oxytocin—a hormone responsible for well-being and love [36], making the individual feel happier and, as such, improving task performance. Barker et al. [55] showed that pets influence their owners through basic interactions such as observing and caring, which helps them to deal effectively with their daily tasks. Gee et al. [56], in an experimental study showed that performance on a memory task was better in the presence of a dog (compared to the absence, or the presence of a person). Therefore, the presence and interaction with pets, during work, when individuals are emotionally attached to them, can improve self-reported job performance. Attachment theory and the COR theory help explain these beneficial effects to workers. Attachment theory [57,58,59] suggests that a close emotional attachment between a pet and an individual provides psychological security, a source of social support, and advanced performance, for the individual, especially during this time of pandemic [60,61]. In addition, the COR theory [2] helps explain employees’ personal gains from working with animals. Accordingly, pets may be viewed as a resource for the individual, thereby promoting attention to the tasks and improving their perceived performance, even though the pandemic times are being lived.

In this study, we focus on affect as a mechanism to explain how teleworking impacts self-reported job performance and we show that this is more beneficial for individuals who work closer to their pets, and when they hold an emotional bond with them. Thus, a day is not only better with a pet, but a pet also makes it a productive day.

### 6.1. Limitations and Future Research

Despite the positive features of this study, such as being a preliminary study in a relevant field, and with two working samples, it has some limitations. First, we must consider the differential sizes between each group (pet-owners and non-pet owners). The non-pet owner group was smaller than the other group; hence, the interpretation of the means comparison results should be regarded with some caution. Second, we used self-reported measures, which might account for common method variance [49,50,51,52,53,54,55,56,57,58,59,60,61,62], however, as referred before, we took some strategies to minimize it. Second, there are studies demonstrating that individual differences (e.g., personality traits) may influence how individuals perceive themselves, for instance regarding performance [63]. For instance, positive affectivity might positively influence self-perceived performance. Thus, future studies should examine whether positive affectivity or other personality traits (e.g., optimism) might influence perceived performance. Additionally, future studies could use other sources of information (e.g., colleagues, supervisors) regarding performance. Third, the fact that data was collected cross-sectional is a limitation. Therefore, future studies could replicate this study through a longitudinal or daily study. These designs would also safeguard type 1 errors—a safeguard to the internal validity of the study. Fourth, we only measured self-reported task performance because we were interested in this specific type of behavior. However, future studies might consider exploring contextual performance or creativity. Fifth, given the period of data collection—in mandatory confinement—there might have been some affect bias as people have experienced dramatic changes in affect, well-being, and mental health [64,65]. Thus, future studies should retest the model. Moreover, we did not measure some pet-related variables, such as the age of pets, and duration of pet ownership because we did not want a too long survey; however, we acknowledge that these variables might have some effect on diverse criterion variables such as performance or well-being. Thus, future studies would consider including such information.

These results open the way for future studies. First, the finding that pets are a condition through which telecommuting impacts affect and self-reported job performance, is relevant, as most studies have disregarded the importance of pets for human and organizational life [9]. Second, it would be interesting to test the model with other criterion variables, for instance, overall health. To do this, future studies could use objective measures of health, such as heart rate or blood pressure. Third, future studies should explore the role of different pet species (e.g., dogs, cats) because there are studies that demonstrated that different species had different effects. For instance, a study developed in a dentist‘s office showed that an aquarium full of fishes provided a relaxing climate and made the space calmer [54]. A study at Ferrari revealed that a cat interacting with people provided little distraction and, at the same time, lowered stress levels [66]. Similarly, diverse studies developed in technology companies showed that dogs interacted more, and needed other types of caring and attention, but could make the environment more dynamic, creative, and warming [30]. Fourth, future studies could retest the model and compare it between participants who own pets, and who do not. Moreover, because many workers were in mandatory remote work, future research should replicate this model, once the pandemic is over, to see if the results are the same. At last, in this study we only explored the benefits of being closer to pets regarding performance; however, it is possible that other pet-side benefits arise during their owners’ teleworking, which strengthens the pet-human attachment. Thus, future studies might consider exploring other benefits for individuals (e.g., health). Plus, it would be relevant to analyze specific behavioral characteristics of pets that can impact human-animal interaction. For example, too much closeness and attachment to pets might be a distracting factor, decreasing one‘s working performance. As such, designing an experience sampling method would be relevant to analyzing daily fluctuations in human-animal interactions and subsequent distractions.

### 6.2. Practical Contributions

In sum, telework and affect are important variables for the prediction of performance. This study also emphasizes that this relationship is stronger when individuals work closer to their pets and to whom emotional attachment is higher. Thus, the relevance of pets at work has important implications for organizational theories and applied purposes, such as performance management, and employee development.

The results show that telecommuting is a way to assist workers’ affective well-being and their self-reported job performance. Thus, adopting this model of work can be a strategy not only for pandemic times but also for the future. This strategy might be particularly important for workers with pets and with high levels of attachment to them.

However, it will not always be possible to have workers working from home (e.g., a hairstylist). In those cases, the presence of pets, as well as other practices related to them, seem to be relevant in organizations, to satisfy the needs of employees and their customers, and at the same time deliver benefits to organizations. It has been argued that the implementation of pet-friendly practices has reduced organizational costs, especially when compared to the benefits it has [46,67]. These benefits assert themselves even in the face of challenges related to health, safety, cultural issues, fears, phobias, and interruptions in the work environment [68,69]. However, practices must be implemented as baby steps. For instance, it should be a starting point to create a “pet day”, which is an open day, in which workers and customers could take their pets to work. Another measure could include a license of bereavement following a pet death or allowing the owner to take their pet‘s birthday off. Other measures could include the extension of family-friendly practices to include pets. For instance, many organizations have aids for their workers’ children‘s education. However, it could be extended to pet caring or to pets’ daycare.

Given the importance associated with positive affect, managers can benefit from acknowledging its relevance for performance. Thus, they should create conditions for their workers to experience more frequently positive affect, for example, giving specific times to workers to make their task breaks, creating specific ways to regularly give feedback to them, and also creating a time and space for them to share it with each other.

## 7. Conclusions

Overall, this study shows that perceived quality of life while telecommuting is positively related to positive affect and performance and seems to be moderated by the pet‘s closeness and attachment. Specifically, this study sheds light on the power that pets play in this path, evidencing the positive interaction between pets’ closeness and emotional attachment in the mediating path.

## Figures and Tables

**Table 1:** Correlations and descriptive statistics of the variables under study.

Variables	*M*	*SD*	1	2	3	4	5	6	7
1. Telework	3.20 ^1^	0.51	-						
2. Positive affect	3.11 ^1^	0.69	0.50 **	-					
3. Self-reported job performance	4.05 ^1^	0.54	0.31 **	0.37 **	-				
4. Pet closeness	2.63 ^1^	1.29	0.00	0.01	0.07	-			
5. Pet attachment	3.78 ^1^	0.99	−0.06	−0.06	0.17 **	0.69 **	-		
6. Sex	-	-	0.01	0.13 **	−0.01	−0.20 **	−0.18 **	-	
7. Age	31.87	9.50	0.02	0.07	0.14**	−0.07	−0.12 *	0.12 *	-

*n* = 401; * *p* < 0.05 ** *p* < 0.001; ^1^ 5-point Likert scale: attitudes toward telework and pet attachment: 1 = totally disagree; 5 = totally agree; positive affect and pet closeness: 1 = never, 5 = always; self-reported job-performance: 1 = very little; 5 = a great deal).

**Table 2:** Means comparisons between the two groups of participants (pet owners versus non-pet owners).

Groups	Pet-Owners	Non-Pet Owners	*F*
Variables	*M* (*SD*)	*M* (*SD*)	
Perceived telework effects	3.30 (0.46)	3.20 (0.45)	4.80 ***
Positive affect	3.21 (0.54)	3.11 (0.73)	4.27 **
Performance	4.10 (0.55)	3.98 (0.45)	3.39 *

Note. Groups: Pet-owners (*n =* 320); non-pet owners (*n =* 81). * *p* < 0.05, ** *p* < 0.01, *** *p* < 0.001.

**Table 3:** Summary regression table of the mediation model (Hypothesis 2).

Model	Positive Affect (M)			Self-Reported Performance (Y)		
	*B*	*SE*	*t*	*B*	*SE*	*T*
Telework (X)	0.67 **	0.07	10.07	0.18 **	0.06	2.92
PA (M)	-	-	-	0.22 **	0.05	4.75
Age	0.00	0.00	0.89	0.01 *	0.00	2.30
Sex	0.17 *	0.07	2.48	−0.07	0.06	−1.21
Indirect Effect	Effect (γ)	BootSE		LLCI-ULCI		
PA	0.15	0.04		[0.08, 0.22]		

Note. *n =* 320; * *p* < 0.05 ** *p* < 0.001. *B* = Unstandardized coefficients; PA = Positive affect.

**Table 4:** Summary regression table of the moderated-moderated-mediation model (Hypothesis 3).

Model	Positive Affect (M)			Self-Reported Performance (Y)		
	*B*	*SE*	*T*	*B*	*SE*	*t*
Telework (X)	0.66 **	0.07	10.07	0.24 **	0.06	3.31
PA (M)	-	-	-	0.13 *	0.06	2.16
Pet closeness (Mod)	-	-	-	−0.19 *	0.08	−2.48
Pet attachment (Mod)	-	-	-	0.19 **	0.04	4.57
PA * Pet attachment * Pet closeness	-	-	-	0.39 **	0.12	2.46
Age	0.00	0.00	0.89	0.01 *	0.00	2.77
Sex	0.17 *	0.07	2.48	−0.02	0.05	−0.43
Index of mod-mod-med effect	Effect (γ)	BootSE		LLCI-ULCI		
PA	0.26	0.14		[0.02, 0.52]		
*R*^2^ = 0.26 *F*_(11,308)_ = 10.45, *p* = 0.00, Δ*R*^2^ = 0.02, *p* = 0.01						

Note. *n =* 320; * *p* < 0.05 ** *p* < 0.001. *B* = Unstandardized coefficients; PA = Positive affect.

## Data Availability

Data will be made available upon reasonable request.

## Appendix A

### Appendix A.1. Survey

#### Appendix A.1.1. The Attitude toward Telework

Please indicate your agreement or otherwise to the statements below

#### Appendix A.1.2. Effectiveness/Productivity

1. When e-working I can concentrate better on my work tasks.
2. E-working makes me more effective to deliver against my key objectives and deliverables.
3. If I am interrupted by family/other responsibilities whilst e-working from home, I still meet my line manager‘s quality expectations.
4. My overall job productivity has increased by my ability to e-work remotely/from home.

#### Appendix A.1.3. Organizational Trust

1. My organisation provides training in e-working skills and behaviours.
2. I trust my organisation to provide good e-working facilities to allow me to e-work effectively.
3. My organisation trusts me to be effective in my role when I e-work remotely.

#### Appendix A.1.4. Interference between Personal and Work-Life

1. My social life is not poor when e-working remotely.
2. My e-working does not take up time that I would like to spend with my family/friends or on other non-work activities.
3. When e-working remotely I do not often think about work related problems outside of my normal working hours.
4. I am happy with my work-life balance when e-working remotely.
5. Constant access to work through e-working is not very tiring.
6. I do not feel that work demands are much higher when I am e-working remotely.
7. When e-working from home I do not know when to switch off/put work down so that I can rest (R).

#### Appendix A.1.5. Flexibility

1. My work is so flexible I could easily take time off e-working remotely, if and when I want to.
2. My line manager allows me to flex my hours to meet my needs, providing all the work is completed.
3. My supervisor gives me total control over when and how I get my work completed when e-working.
4. Scale (1—totally disagree; 5—totally agree)

#### Appendix A.1.6. Positive Affect

Today, please indicate below approximately how often you have felt the following while you were working in your job. Everyone has a lot of overlapping feelings, so you’ll have a total for all the items that are much greater than 100% of the time.

1. Enthusiastic.
2. Calm.
3. Joyful.
4. Relaxed.
5. Inspired.
6. Laid-back.
7. Excited.
8. At ease.

Scale (1) never to (5) always

#### Appendix A.1.7. Self-Reported Job Performance

On the last day (you worked), how well were you

1. Today, I achieved my job goals.
2. Today I made the right decisions.
3. Today I permed without mistakes.
4. Today I handled the responsibilities and daily demands of my work.
5. Today I get the things done.
6. Today I get along with others at work.

Scale (1) very little (5) a great deal

#### Appendix A.1.8. Emotional Attachment to Pets

Please consider the animal you have lived with the longest, and answer using the following criteria. (1) completely disagree to (5) completely agree.

1. My pet knows when I’m feeling bad.
2. I often talk to other people about my pet.
3. My pet understands me.
4. I believe that loving my pet helps me stay healthy.
5. My pet and I have a very close relationship.
6. I play with my pet quite often.
7. I consider my pet to be a great companion.
8. My pet makes me feel happy.
9. I am not very attached to my pet (R).
10. Owning a pet adds to my happiness.
11. I consider my pet to be a friend.

#### Appendix A.1.9. Physical Closeness to Pets

Please, think about the last day you were working remotely. Indicate the frequency through which the following situations with your pet occurred during the working day.

1. In telework, I usually take breaks to interact with my pet.
2. While you work from home, my pet is close to me when I am working.
3. During telework, my pet is not close to me while I work (R).
4. While I am working, I use to interact with my pet.

Scale (1) never to (5) always.
